# *KRAS*-G12C Mutation in One Real-Life and Three Population-Based Nordic Cohorts of Metastatic Colorectal Cancer

**DOI:** 10.3389/fonc.2022.826073

**Published:** 2022-02-16

**Authors:** Emerik Osterlund, Ari Ristimäki, Soili Kytölä, Teijo Kuopio, Eetu Heervä, Timo Muhonen, Päivi Halonen, Raija Kallio, Leena-Maija Soveri, Jari Sundström, Mauri Keinänen, Annika Ålgars, Raija Ristamäki, Halfdan Sorbye, Per Pfeiffer, Luís Nunes, Tapio Salminen, Annamarja Lamminmäki, Markus J. Mäkinen, Tobias Sjöblom, Helena Isoniemi, Bengt Glimelius, Pia Osterlund

**Affiliations:** ^1^Department of Immunology, Genetics and Pathology, Uppsala University, Uppsala, Sweden; ^2^Department of Pathology, HUSLAB, HUS Diagnostic Center, Helsinki University Hospital, Helsinki, Finland; ^3^Applied Tumor Genomics Research Program, Research Programs Unit, Faculty of Medicine, University of Helsinki, Helsinki, Finland; ^4^Department of Genetics, HUSLAB, HUS Diagnostic Center, Helsinki University Hospital, Helsinki, Finland; ^5^Department of Genetics, University of Helsinki, Helsinki, Finland; ^6^Department of Pathology, Central Finland Hospital Nova, Jyväskylä, Finland; ^7^Department of Biological and Environmental Science, University of Jyväskylä, Jyväskylä, Finland; ^8^Department of Oncology, Turku University Hospital, Turku, Finland; ^9^Department of Oncology, University of Turku, Turku, Finland; ^10^Department of Oncology, South Carelia Central Hospital, Lappeenranta, Finland; ^11^Department of Oncology, University of Helsinki, Helsinki, Finland; ^12^Department of Oncology, Helsinki University Hospital, Helsinki, Finland; ^13^Department of Oncology, Oulu University Hospital, Oulu, Finland; ^14^Department of Oncology, University of Oulu, Oulu, Finland; ^15^Home Care, Geriatric Clinic and Palliative Care, Joint Municipal Authority for Health Care and Social Services in Keski-Uusimaa, Hyvinkää, Finland; ^16^Department of Pathology, Turku University Hospital, Turku, Finland; ^17^Institute of Biomedicine, University of Turku, Turku, Finland; ^18^Department of Genetics, Fimlab Laboratories, Tampere, Finland; ^19^Department of Oncology, Haukeland University Hospital, Bergen, Norway; ^20^Department of Clinical Science, University of Bergen, Bergen, Norway; ^21^Department of Oncology, Odense University Hospital, Odense, Denmark; ^22^Department of Clinical Research, University of Southern Denmark, Odense, Denmark; ^23^Department of Oncology, Tampere University Hospital, Tampere, Finland; ^24^Department of Oncology, University of Tampere, Tampere, Finland; ^25^Department of Oncology, Kuopio University Hospital, Kuopio, Finland; ^26^Department of Medicine, University of Eastern Finland, Kuopio, Finland; ^27^Department of Pathology, Oulu University Hospital, Oulu, Finland; ^28^Department of Pathology, Cancer and Translational Medicine Research Unit, University of Oulu, and Medical Research Center Oulu, Oulu, Finland; ^29^Department of Transplantation and Liver Surgery, Helsinki University Hospital, Helsinki, Finland; ^30^Department of Surgery, University of Helsinki, Helsinki, Finland; ^31^Department of Gastrointestinal Oncology, Karolinska Universitetssjukhuset, Stockholm, Sweden; ^32^Department of Oncology/Pathology, Karolinska Institutet, Stockholm, Sweden

**Keywords:** colorectal cancer, metastatic, *KRAS* mutation, *KRAS-*G12C mutation, population-based, real-world

## Abstract

**Background:**

*KRAS* mutations, present in over 40% of metastatic colorectal cancer (mCRC), are negative predictive factors for anti-EGFR therapy. Mutations in *KRAS-*G12C have a cysteine residue for which drugs have been developed. Published data on this specific mutation are conflicting; thus, we studied the frequency and clinical characteristics in a real-world and population-based setting.

**Methods:**

Patients from three Nordic population-based cohorts and the real-life RAXO-study were combined. *RAS* and *BRAF* tests were performed in routine healthcare, except for one cohort. The dataset consisted of 2,559 patients, of which 1,871 could be accurately classified as *KRAS*, *NRAS*, and *BRAF*-V600E. Demographics, treatments, and outcomes were compared using logistic regression. Overall survival (OS) was estimated with Kaplan–Meier, and differences were compared using Cox regression, adjusted for baseline factors.

**Results:**

The *KRAS-*G12C frequency was 2%–4% of all tested in the seven cohorts (mean 3%) and 4%–8% of *KRAS* mutated tumors in the cohorts (mean 7%). Metastasectomies and ablations were performed more often (38% vs. 28%, p = 0.040), and bevacizumab was added more often (any line 74% vs. 59%, p = 0.007) for patients with *KRAS-*G12C- vs. other *KRAS*-mutated tumors, whereas chemotherapy was given to similar proportions. OS did not differ according to *KRAS* mutation, neither overall (adjusted hazard ratio (HR) 1.03; 95% CI 0.74–1.42, reference *KRAS*-G12C) nor within treatment groups defined as “systemic chemotherapy, alone or with biologics”, “metastasectomy and/or ablations”, or “best supportive care”, *RAS* and *BRAF* wild-type tumors (n = 548) differed similarly to *KRAS*-G12C, as to other *KRAS*- or *NRAS*-mutated (n = 66) tumors.

**Conclusions:**

In these real-life and population-based cohorts, there were no significant differences in patient characteristics and outcomes between patients with *KRAS-*G12C tumors and those with other *KRAS* mutations. This contrasts with the results of most previous studies claiming differences in many aspects, often with worse outcomes for those with a *KRAS-*G12C mutation, although not consistent. When specific drugs are developed, as for this mutation, differences in outcome will hopefully emerge.

## Introduction

Mutations in the *RAS* genes are common oncogenic drivers (1). In colorectal cancer (CRC), Kirsten rat sarcoma viral oncogene homolog (*KRAS*) is mutated (mt) in over 40% and neuroblastoma RAS viral oncogene homolog (*NRAS*) in about 4% (2). The specific mutated codon impacts the activity of the enzyme, and this may impact the clinical behavior of the tumor. Activating missense mutations in *RAS* cause resistance to epidermal growth factor receptor (EGFR) inhibition, and although different mutations may have somewhat variable resistance (3, 4), in practice, all *KRAS* and *NRAS* mutations are predictive markers for no benefit of anti-EGFR therapy. It has been challenging to develop therapies directed against *KRAS* mutated tumors due to multiple different activating mutations in the RAS genes (5, 6). The *KRAS-*G12C has a cysteine residue for which specific drugs, such as sotorasib (AMG 510, Amgen, Thousand Oaks, CA, USA) and adagrasib (MRTX849, Mirati Therapeutics, San Diego, CA, USA), have been developed and clinically explored with promising results (7, 8).

The importance of different *KRAS* mutations on the clinical behavior of metastatic CRC (mCRC) has been explored, and differences have been reported (9). The clinical relevance of *KRAS-*G12C is unclear (10–17). The frequency of *KRAS-*G12C has been reported to be 2%–8% in large databases of molecularly analyzed CRCs (12, 14, 18, 19). In hospital-based series, the proportion of *KRAS-*G12C tumors was 2%–4% of all mCRC tumors (11, 15, 16, 20), whereas greater variability was seen in the proportion of the *KRAS-*mutated population (6%–17%) (11, 13, 16, 17, 20–22). Sex- and age-related differences and differences in the metastatic pattern have been reported between different *KRAS* mutations, with no consistent results (12, 13, 22).

Several studies have explored the relation between progression-free (PFS) and overall survival (OS) and *KRAS* mutation type in mCRC. Two Italian hospital-based series reported worse survival for those with *KRAS-*G12C and *KRAS*-G12S compared to other *KRAS* mutations (13, 20). In a pooled analysis of three German trials of retrospectively analyzed *KRAS* codon 12 mutant tumors, patients with G12C (and G12S) mutations fared worse when treated with chemotherapy and EGFR inhibition (23). In a follow-up study including two more trials, the same group found no difference in PFS or OS between different *KRAS* exon 2 mutations, but *KRAS-*G12C had the numerically worst median OS (mOS) and was the only mutation that significantly differed from *KRAS* wild-type (wt) tumors (11). Among 4,632 patients with molecularly profiled tumors at MD Anderson Cancer Center, 134 (3%) had a *KRAS-*G12C mutation. When 53 additional patients were added, those with a *KRAS-*G12C had worse PFS and OS (15). A Japanese study including 1632 patients treated with chemotherapy at 4 hospitals found 45 (6%) *KRAS-*G12C tumors among 696 *KRAS* mutated tumors and similarly found that both PFS and OS were worse for the *KRAS-*G12C cases compared to those having other *KRAS* mutations (16). Finally, in yet another Italian hospital-based study, 120 patients with *KRAS* mutated tumors were treated with a doublet chemotherapy regimen and bevacizumab; 15 (12%) patients with a *KRAS-*G12C had a lower response rate than those with another mutation (27% vs. 52%, p = 0.017); however, no differences in PFS or OS were seen (17).

The studies performed so far have been restricted to patients included in clinical trials, hospital-based series, or large databases with unknown patient selection. Since certain molecular changes in the background population of patients with mCRC differ substantially from those in clinical trials and hospital series (24–26), the representativity of the present knowledge can be discussed. Further, since these mutations are not particularly common, the number of cases is limited in most studies, making estimates uncertain. Extensive knowledge about *KRAS-*G12C frequency, the possible presence of characteristics differing from other *KRAS* mutations, and their relation to survival and treatment response is not available. For these reasons, we examined three population-based Nordic patient materials and one large real-life material for the presence of *KRAS-*G12C and their relations to patient characteristics and outcome compared to those having other *RAS* and v-Raf murine sarcoma viral oncogene homolog B (*BRAF*)-V600E mutations.

## Materials and Methods

**Figure 1:**
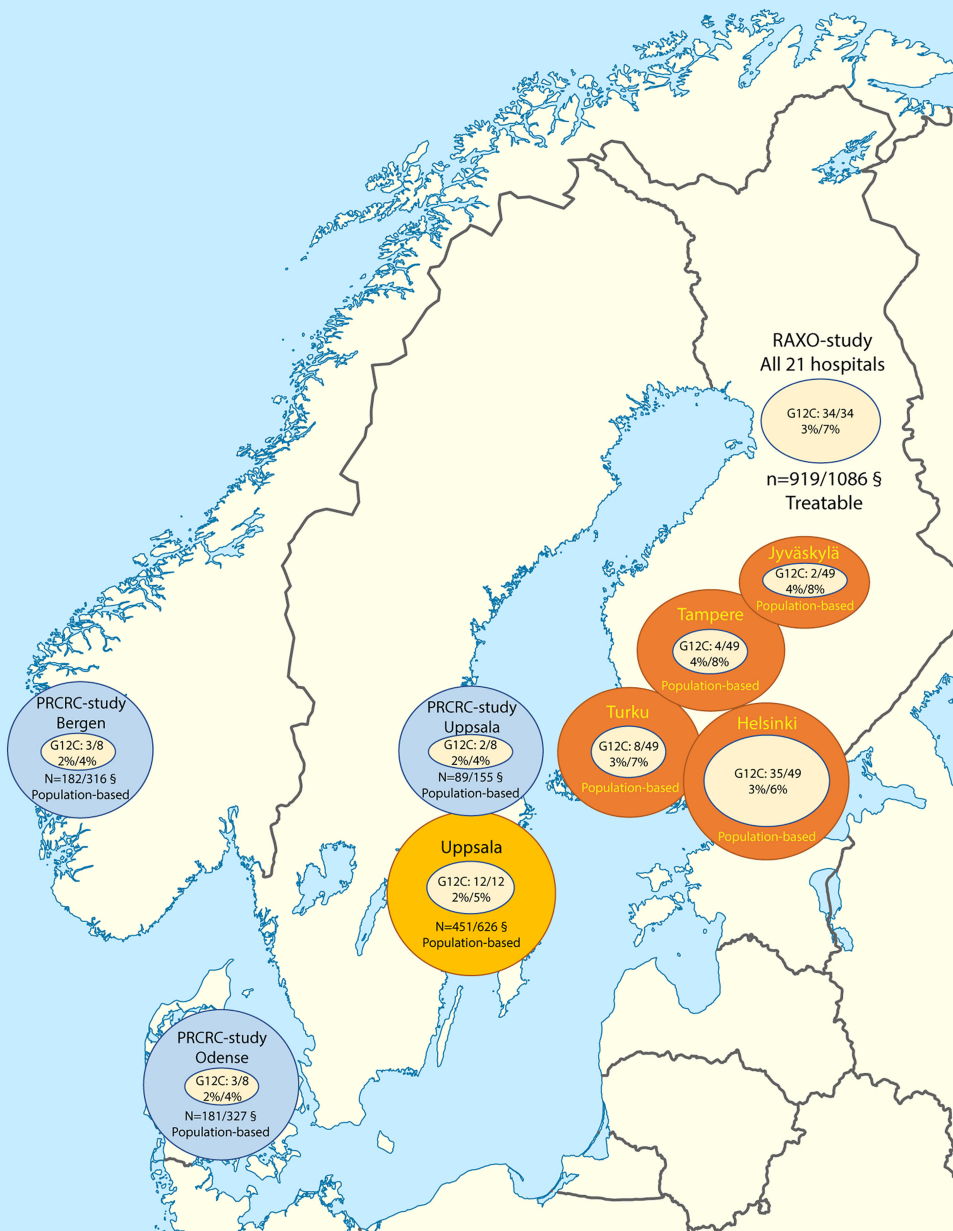
Patient inclusion in the Nordic cohorts including *KRAS*-G12C mutation rates per all tested (at least *KRAS*) tumors and among all *KRAS* **-**mutated tumors.^§^ Adequately grouped *KRAS*, *NRAS*, and *BRAF-*V600E status/study patients.

Patients from four Nordic cohorts were included: the prospective real-life Finnish RAXO-study, a population-based data collection cohort of Finnish patients molecularly tested at four hospitals covering all medical care in the surrounding regions (Helsinki, Jyväskylä, Tampere, and Turku), the population-based Scandinavian prospective registration of mCRC (PRCRC-study), and a Swedish population-based cohort (Uppsala region) (**Figure 1** and **Supplementary Table 1**).

Inclusion criteria for the RAXO-study (27, 28) were patients eligible for first-line chemotherapy with any oncological treatment regimen, age over 18 years, and histologically confirmed CRC with distant metastases or locally advanced primary tumor not curatively treatable. The patients were recruited between 2012 and 2018. In the Finnish population-based RAXO data-collection cohort (referred to here as the Finnish population cohort), all patients with a diagnosis of mCRC between 2011 and 2018 from four regions (Helsinki, Jyväskylä, Tampere, and Turku, covering 62% of the Finnish population) were included [the prospective RAXO data-collection protocol and preliminary results from Tampere and Turku have been published (27)]. This cohort (n = 3,953) partly overlaps with the real-life RAXO-study (n = 1,060) for mutation frequencies; most of the hospitals recruiting patients to the real-life RAXO study were in the four Finnish regions. The exact frequency of *KRAS*-G12C mutations among all tested tumors could not be calculated since validated comprehensive data of all non-*KRAS-*G12C cases at these four hospitals are not available; therefore, we have not included detailed characteristics of these patients in the presentation, only the *KRAS-*G12C cases (see **Supplementary Table 2**). Duplicate patients with *KRAS-*G12C tumors from the RAXO-study were removed (n = 23) from the Finnish population cohort.

In the PRCRC-study, all patients (n = 798) with a diagnosis of incurable metastatic disease from three regions in Norway, Denmark, and Sweden were included between 2003 and 2006 (29). In the Uppsala region cohort, all mCRC patients were prospectively identified in a biobank initiative Uppsala-Umeå Comprehensive Cancer Consortium (UCAN)) (30) since April 2010, and the remaining patients with mCRC since January 2010 were retrospectively identified using a hospital-based registry and the Swedish ColoRectal Cancer Registry (SCRCR) (31). After a validation study against the medical records of all patients with a diagnosis of CRC since January 2010, this cohort can be considered 100% complete (32).

The patient cohorts are presented in **Figure 1**. To compare characteristics of patients with *KRAS-*G12C tumors with those other *KRAS* mutated from the same populations, demographics for the real-life RAXO-study, the PRCRC-study, and Uppsala region cohorts, excluding those identified in the Finnish population cohort (from the four Finnish regions outside the real-life RAXO study), are presented in **Supplementary Tables 4, 5**.

The patients were treated according to routine clinical practice during the inclusion periods. Details for the oldest PRCRC cohort have been described in (29). For the other more recent cohorts, the European Society for Medical Oncology (ESMO) guidelines were followed (33, 34).

### Molecular Analyses

Testing for the presence of *RAS* and *BRAF* mutations and mismatch repair analyses in the real-life RAXO-study, the Finnish population cohort, and the Uppsala region cohort was done in clinical routine (or within a study program for 14 patients in the Uppsala region cohort) using accredited techniques in the majority, as described below. In the Uppsala region, the ambition was to test for these mutations in all patients, whereas the indication to test in the RAXO-study and the Finnish population cohort was planned systemic therapy for mCRC. The techniques used for testing varied through the years. During the first years of inclusion, a pyrosequencing technique was usually performed for Swedish cohorts (35) and in 0.3% in the RAXO-study, or reverse transcriptase polymerase chain reaction (RT-PCR) for Finnish cohorts (42% in the RAXO-study), but Sanger PCR was rarely used (0.7% of RAXO-study). Two-step Biocartis Idylla testing (first *KRAS* [exons 2–4] testing and if wt then *NRAS* [exons 2–4] and *BRAF* [V600E and non-V600E] testing) has been used for 123 (12%) patients in the RAXO-study at Oulu and Turku University Hospitals. During the latter parts or at least from 2014, a next-generation sequencing (NGS) technique was generally used. The composition of the NGS panels varied between the hospitals and through the years but always contained analyses, allowing testing of the presence of hotspot mutations in extended *RAS*, including both *KRAS* and *NRAS* exons 2–4 (codons 12, 13, 59, 61, 117, and 146) and for the *BRAF*-V600E mutation according to ESMO recommendations (34). Routine NGS testing was performed in the molecular pathology units at Uppsala, Helsinki, Tampere, Jyväskylä, Charité, and Nijmegen University Hospitals. In the PRCRC-study cohort, these analyses were performed using an NGS panel as described in (26). All patients with at least *KRAS* exon 2 testing were included in frequency analyses, presented as *KRAS-*G12C rate of all tested tumors and of patients with any *KRAS* mutation.

Patients that could not be accurately classified for *KRAS*, *NRAS*, and *BRAF* were excluded from demographics presentation and survival analyses (**Figure 1** and **Supplementary Tables 1, 2**). To be classified as adequately analyzed, the presence of a *KRAS* or *BRAF*-V600E mutation, assuming that these mutations are mutually exclusive, was sufficient. Before a tumor was considered *RAS* and *BRAF* wt, testing of *KRAS* exons 2–4, *NRAS* exons 2–4, and *BRAF*-V600E was required. In 428 (17%) of 2,559 patients, no molecular tests had been performed, and 260 (10%) could not be adequately characterized (due to missing *KRAS*, *NRAS*, or *BRAF* analyses in 229, atypical non-*BRAF*-V600E mutations in 24, and multiple mutations in 7 [*KRAS* and *NRAS* (n = 6) and *KRAS-*G12C and *BRAF*-V600E (n = 1)]. OS is presented separately for the tested but not adequately characterized group.

### Statistical Analyses

Demographics, treatments, and outcomes between *KRAS-*G12C and other *KRAS* mutations were compared with logistic regression models and with chi-square for cohorts. The Mann–Whitney test was used to compare non-normally distributed variables. Survival was estimated with the Kaplan–Meier estimate and compared using the log-rank test and Cox regression. OS was also adjusted for age, sex, Eastern Cooperative Oncology Group (ECOG) performance status, primary tumor site, presentation and number of metastatic sites, treatment groups, and cohort. *KRAS-*G12C was used as a reference in these analyses. Two-tailed p-values of <0.05 and 95% CI not crossing 1 were considered statistically significant. All statistical analyses were performed using SPSS Statistics version 25 and 27.

### Clinical Trial Identification and Ethical Permission

Clinical trial identification is NCT01531595 and EudraCT 2011-003137-33 for the RAXO-study and Finnish population cohort. Ethical permission was obtained for all collections by the ethical committees at the Helsinki University Hospital, Haukeland University Hospital, Odense University Hospital, and Uppsala University. These permissions included retrospective identification of all patients with a diagnosis of mCRC living in the catchment areas of the Scandinavian PRCRC cohort and in the Uppsala region at the time of diagnosis of their primary tumor and to perform the molecular analyses of their tumors. All studies were conducted in accordance with the Declaration of Helsinki.

## Results

### Characteristics of All and Tested Patients in the Cohorts

The characteristics of all 2,559 patients in the cohorts are presented in **Supplementary Table 1**. Patients in the PRCRC-study were slightly older, were more likely female, had more right-sided colon cancers, and were cared for without any tumor controlling therapy (best supportive care (BSC)) more often than those in the more recent Uppsala region and RAXO-study cohorts. Patients in the real-life RAXO-study were younger, had better ECOG performance status, and more often received active tumor-controlling therapy (according to inclusion criteria), especially metastasectomies and/or local ablative therapy (LAT). mOS of all patients in the cohorts was the shortest in the oldest PRCRC-study cohort, intermediate in the recent population-based cohorts [Uppsala region 15.3 months, **Supplementary Table 1**, Tampere region 16 months, and Turku region 16 months, data shown in (27)], and the longest in the real-life RAXO-study. The OS differences were much smaller when analyzed per treatment group, i.e., in patients receiving cytotoxics only, cytotoxics combined with anti-vascular endothelial growth factor (anti-VEGF) or anti-EGFR, and metastasectomy and/or LAT (such as thermoablation and stereotactic radiotherapy) (**Supplementary Table 1**).

Of all molecularly tested (at least *KRAS* exon 2 analyzed) tumors in the cohorts, between 2% and 4%, with a mean of 3%, had a mutation in *KRAS*-G12C (PRCRC-study 2%, Uppsala region 2%, Helsinki region 3%, real-life RAXO-study 3%, Turku region 3%, Jyväskylä region 4%, and Tampere region 4%; **Figure 1**).

The proportion of a *KRAS-*G12C mutation among the tumors with a *KRAS* mutation was between 4% and 8%, with a mean of 7% (PRCRC-study 4%, Uppsala region 5%, Helsinki region 6%, RAXO-study 7%, Turku region 7%, Jyväskylä region 8%, and Tampere region 8%; **Figure 1**). *KRAS-*G12C was mutually exclusive for all but one patient (with *BRAF*-V600Emt).

### Characteristics of Molecularly Accurately Grouped Patients

The characteristics of all 1,871 patients accurately characterized as *KRAS*, *NRAS*, and *BRAF*-V600E from the four cohorts including the 49 unique Finnish population cohort patients identified with a *KRAS-*G12C in the four hospital regions in Finland are presented in **Figure 1** and **Supplementary Table 2**. **Supplementary Table 2** shows that patients accurately analyzed for *KRAS*, *NRAS*, and *BRAF* were more similar than all patients in the cohorts were (presented in **Supplementary Table 1**). The proportions of the different treatment groups differed between the cohorts, and OS accordingly. The OS did not differ between cohorts in the treatment groups, i.e., cytotoxics only, cytotoxics combined with anti-VEGF/anti-EGFR, or metastasectomy and/or LAT (**Supplementary Table 2**). The BSC group had the shortest OS in all cohorts, although it was longer in the population cohorts compared to the real-life RAXO study.

The rates of different mutations in accurately molecularly analyzed patients are shown in **Supplementary Table 2**. The proportion of tumors with a *KRAS* and *NRAS* mutation was rather similar between the cohorts. The most striking difference between the cohorts was in the proportion of *BRAF*-V600E-mutated tumors: 20% in the PRCRC-study, 20% in the Uppsala region, 18% in the Finnish population cohort, and 10% in the real-life RAXO-study.

The characteristics of the patients with the different *RAS* and *BRAF*-V600E mutations are shown in **Table 1** and **Supplementary Table 3**. The same characteristics for the patients excluding the 49 patients from the Finnish population cohorts are shown in **Supplementary Table 4**. No differences were observed between the patients with *KRAS-*G12C (n = 103) or other *KRAS* mutations (n = 881) according to age, sex, performance status, smoking status, synchronous/metachronous presentation, primary tumor site, degree of differentiation, histology, number of metastatic sites, metastatic location, blood counts, alkaline phosphatase, or carcinoembryonic antigen levels. *KRAS* mutated tumors were seldom associated with deficient mismatch repair (dMMR) status (3% for both *KRAS*-G12C and other *KRAS*mt), however, based upon a few cases; in contrast, dMMR was associated with a *BRAF*-V600E mutation much more frequently (29%). For *KRAS-*G12C vs. other *KRAS* mutations, the primary tumor sites were the right colon in 28% vs. 34%, the left colon in 35% vs. 30%, and the rectum in 37% vs. 36%. Liver metastases were present in 64% vs. 70%, lung in 39% vs. 36%, distant lymph nodes in 24% vs. 23%, and peritoneal in 15% vs. 19% of *KRAS-*G12C compared with other *KRAS* mutations.

**Table 1:** Demographics according to the type of mutation.

		All		*KRAS*-G12C mt		Other *KRAS* mt		*NRAS* mt		*RAS* and *BRAF* wt		*BRAF-*V600E mt		p-Value*
Total		1,871	100%	103	100%	881	100%	66	100%	548	100%	273	100%	
Median age (range)		68	(22–99)	67	(35–86)	69	(23–99)	64	(30–87)	67	(22–95)	70	(33–90)	**0.036**
Age groups	≤70	1,074	57%	63	61%	484	55%	44	67%	350	64%	137	50%	ref
	>70	797	43%	40	39%	397	45%	22	33%	198	36%	136	50%	0.230
Sex	Female	818	44%	46	45%	378	43%	28	42%	198	36%	168	62%	ref
	Male	1,053	56%	57	55%	503	57%	38	58%	350	64%	105	38%	0.734
ECOG Performance status	0	593	32%	27	26%	297	34%	25	38%	176	32%	68	25%	ref
	1	815	44%	50	49%	394	45%	31	47%	241	44%	99	37%	0.184
	2-4	457	25%	25	25%	188	21%	10	15%	130	24%	104	38%	0.194
	Missing	6	–	1	–	2	–	0	–	1	–	2	–	–
Primary tumor	Right colon	624	34%	29	28%	295	34%	9	14%	95	17%	196	73%	ref
	Left colon	619	33%	36	35%	266	30%	26	39%	241	44%	50	19%	0.225
	Rectum	612	33%	38	37%	312	36%	31	47%	207	38%	24	9%	0.409
	Multiple/unknown	16	–	0	–	8	–	0	–	5	–	3	–	–
Differentiation	Well/moderate	1,194	79%	74	85%	588	84%	47	86%	363	81%	122	53%	ref
	Poor/undifferentiated	325	21%	13	15%	111	16%	8	15%	85	19%	108	47%	0.821
	Missing	352	–	60	–	182	–	11	–	100	–	43	–	–
Presentation of metastases	Synchronous	1,147	61%	61	59%	555	63%	41	62%	318	58%	172	63%	ref
	Metachronous	724	39%	42	41%	326	37%	25	38%	230	42%	101	37%	0.454
Number of metastatic sites	1	916	49%	54	52%	443	50%	31	47%	263	48%	125	46%	ref
	2	615	33%	33	32%	287	33%	22	33%	178	32%	95	35%	0.803
	3+	340	18%	16	16%	151	17%	13	20%	107	20%	53	19%	0.640
Metastatic sites	Liver	1,289	69%	66	64%	615	70%	51	77%	412	75%	145	53%	0.234
	Lung	587	31%	40	39%	321	36%	22	33%	136	25%	68	25%	0.633
	Lymph nodes	499	27%	25	24%	199	23%	18	27%	152	28%	105	38%	0.700
	Peritoneum	388	21%	15	15%	169	19%	11	17%	104	19%	89	33%	0.257
	Bone	69	4%	3	3%	31	4%	2	3%	23	4%	10	4%	0.750
	Other	257	14%	15	15%	106	12%	9	14%	78	14%	49	18%	0.460
MMR-status	pMMR	908	92%	33	97%	425	97%	27	90%	292	98%	131	71%	ref
	dMMR	77	8%	1	3%	12	3%	3	10%	7	2%	54	29%	0.947
	Missing	886	–	69	–	444	–	36	–	249	–	88	–	–

dMMR, deficient mismatch repair; ECOG, Eastern Cooperative Oncology Group; MMR, mismatch repair; pMMR, proficient mismatch repair; PS, performance status.

*p-Value between KRAS-G12C mt and Other KRAS mt. Significant differences in bold.

### Treatments and Outcome

Treatments provided for all accurately molecularly analyzed patients are shown in **Table 2** and **Supplementary Table 5**. Systemic therapy was given to 85% vs. 84% of *KRAS-*G12C vs. other *KRAS* mutant cases, with no differences in the number of lines of therapy, treatment responses, and drug exposures apart from more anti-VEGF use in any line (but not first line) for patients with a *KRAS-*G12C mutation.

**Table 2:** Treatment according to the type of mutation.

		All		*KRAS*-G12C mt		Other *KRAS* mt		p-Value*	*NRAS* mt		*RAS* and *BRAF* wt		*BRAF* V600E mt	
Total		1,871	100%	103	100%	881	100%		66	100%	548	100%	273	100%
Type of treatment	Systemic therapy	1,077	58%	51	50%	510	58%	ref	39	59%	308	56%	169	62%
	Metastasectomy and/or LAT	516	28%	39	38%	246	28%	**0.042**	22	33%	180	33%	29	11%
	Best supportive care	278	15%	13	13%	125	14%	0.904	5	8%	60	11%	75	27%
Total chemotherapy		1,558	100%	88	100%	743	100%		59	100%	476	100%	192	100%
Number of lines	1	625	40%	32	36%	291	39%	ref	21	36%	197	41%	84	44%
	2	433	28%	27	31%	204	27%	0.503	14	24%	115	24%	73	38%
	≥3	500	32%	29	33%	248	33%	0.820	24	41%	164	34%	35	18%
First line chemotherapy	5-FU	1,519	97%	86	98%	729	98%	0.802	57	97%	462	97%	185	96%
	Oxaliplatin	913	59%	48	55%	453	61%	0.245	33	56%	268	56%	111	58%
	Irinotecan	414	27%	26	30%	165	22%	0.124	17	29%	153	32%	53	28%
	Bevacizumab	670	43%	47	53%	348	47%	0.244	33	56%	163	34%	79	41%
	anti-EGFR	164	11%	1	1%	12	2%	0.734	4	7%	136	29%	11	6%
Best response in first line	PR/CR/NED	822	57%	47	57%	368	53%	0.579	32	58%	307	68%	68	40%
	SD	428	30%	23	28%	238	35%	ref	14	25%	97	21%	56	33%
	PD	198	14%	13	16%	83	12%	‘‘	9	16%	48	11%	45	27%
	Missing	110	–	5	–	54	–	–	4	–	24	–	23	–
Chemotherapy all lines	5-FU	1,525	98%	87	99%	731	98%	0.734	57	97%	463	97%	187	97%
	Oxaliplatin	1,193	77%	67	76%	581	78%	0.659	47	80%	355	75%	143	74%
	Irinotecan	1,088	70%	61	69%	511	69%	0.917	46	78%	344	72%	126	66%
	Bevacizumab	866	56%	65	74%	437	59%	**0.007**	37	63%	235	49%	92	48%
	Anti-EGFR	364	23%	0	0%	36	5%	NE	10	17%	289	61%	29	15%

NE, no estimate; ref, reference.

*p-Value between KRAS-G12C mt and Other KRAS mt. Significant differences in bold.

**Figure 2:**
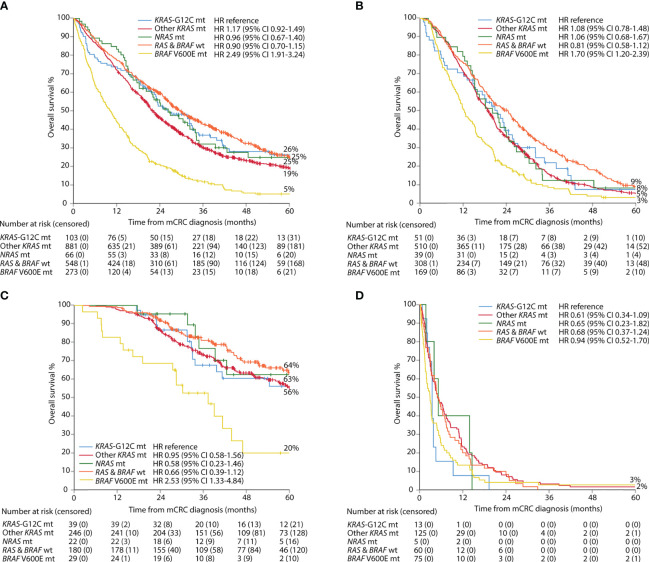
Overall survival per group of mutation: all patients **(A)**, systemic therapy only **(B)**, metastasectomy and/or local ablative therapy **(C)**, and best supportive care **(D)**.

Metastasectomy and LAT were performed in 38% vs. 28% for *KRAS-*G12C vs. other *KRAS* mutant cases (p = 0.042). This did not have a significant effect on OS (median 26.2 vs. 22.0 months; adjusted hazard ratio (HR) 1.03; 95% CI 0.75–1.43, with *KRAS-*G12C as the reference; unadjusted HR shown in **Figure 2A**). OS within each separate treatment group (metastasectomy/LAT, “systemic therapy only” and BSC alone) did not differ (**Figures 2B–D** and **Supplementary Figure 1** for treatment groups in *KRAS-*G12C tumors only). Median PFS in patients treated with “systemic therapy only” was 12.7 months in *KRAS-*G12C tumors and 11.7 months in other *KRAS* mutations, with adjusted HR 1.09 (95% CI 0.71–1.65) (**Supplementary Figure 3A**). OS and PFS excluding the 49 patients from the Finnish population cohort are presented in **Supplementary Figures 2A–D, 3B**.

### Characteristics of Patients With a *KRAS*G12C Mutation in Comparison With Those With Other *RAS* and *BRAF* Mutations

*RAS* and *BRAF* wt tumors revealed the same associations for *KRAS-*G12C as for other *KRAS* or *NRAS* mutations. Patients with tumors being *RAS* and *BRAF* wt were right-sided less often (**Table 1**). Patients with these tumors also had the longest OS (median 29.6 vs. 26.2 months for *KRAS-*G12C and 22.0 months for other *KRAS*mt (**Figure 2A**). Median PFS in patients treated with “systemic chemotherapy only” was longer in *RAS* and *BRAF* wt tumors (10.6 months) than in *KRAS-*G12C tumors (10.1 months), other *KRAS*mt (9.8 months), or *NRAS*mt (9.4 months) (**Supplementary Figure 3A**). mOS in the patients not adequately classified as *RAS* and *BRAF* wt (or with double mutations) was 22.6 months.

*BRAF*-V600E cases differed in many aspects from the other mutations and more often had progressive disease (PD) as the best response to chemotherapy, a surgical procedure was done less often, and the poorest PFS and OS were seen, regardless of treatment group (**Table 1**, **Figures 2A–D**, **Supplementary Tables 3, 4**, and **Supplementary Figures 2, 3**).

## Discussion

In these real-life and population-based cohorts, mutations in *KRAS*-G12C were seen in 2%–4% of all tumors in patients with mCRC; actually, the proportion was the lowest in the population-based cohorts, with 2%–3%, and up to 4% when analyzed in the real-life RAXO-study and Finnish population cohorts, where most of the patients could be actively treated. The proportion of this mutation among *KRAS* mutated tumors was 4%–8%, similarly the lowest in the population-based cohorts (4% in the PRCRC-study and Uppsala region), proportions that are considerably lower than in all so far reported hospital-based or clinical trial series (6%–17%) (11, 13, 15, 16, 20, 21), but in line with proportions seen in large databases of molecularly tested tumors (3%–7%) (12, 14, 18, 36).

We were not able to detect any significant or clinically relevant differences in patient characteristics, treatments provided, and outcomes between the 103 *KRAS-*G12C cases compared to those with other *KRAS* or *NRAS* mutations. This contrasts with the results of previous studies claiming differences in several aspects, however, not consistent. Above all, we could not show any differences in response rates, PFS, or OS, especially not when analyzed per treatment group defined as cytotoxics with or without anti-VEGF/anti-EGFR, or curative intent metastasectomy and/or LAT, allowing us to conclude that tumors with this mutation behave like those with other *KRAS* mutations in mCRC. Any association of *KRAS-*G12C in smokers, whether current or ex-smokers, versus never smokers could not be verified among mCRC patients, in contrast to lung cancer where *KRAS-*G12C has been associated with smoking, especially in women (37, 38). Salem et al. reported that dMMR status was less common in *KRAS-*G12C than in other tumors (36), a trend not verified in our study (3% for both). But we had numerically higher dMMR rates in *NRAS* **-**mutated (10%) and *BRAF*-V600E-mutated (29%) tumors, with the caveat of small patient numbers. *KRAS-*G12C mutations were mutually exclusive for other *KRAS*, *NRAS*, and *BRAF*-V600E mutations in all but one case (with *BRAF*-V600Emt), which is in line with previous findings (14).

To show differences between rare tumor properties, large and preferably non-selected patient series are required. Most studies exploring the clinical relevance of tumor properties stem from clinical trials, and in this respect, randomized trials are considered most valuable. However, inclusion in trials and particularly in randomized trials means that patient selection can be extensive. We and others have previously reported that *BRAF*-V600E mutations are at least twice as common in the background population as in the trial-/hospital-based series (24, 26, 39), and similarly, dMMR is about twice as common (26, 40). When the disease is metastatic, both *BRAF*-V600E and dMMR mean poor prognosis, and the most probable reason behind these prevalence differences is that patients with these tumors do not frequently manage to be included because of rapidly progressing disease. Comorbidities related to older age may also contribute to the risk. A proportion of 2% of *KRAS-*G12C in the population-based cohorts, including a significant BSC population and higher proportions of treatable patients as in the real-life RAXO-study and in the Finnish population cohorts, where only “treatable” patients were molecularly analyzed, rather indicates that, if anything, the prognosis for this mutation is better than that for other *KRAS* mutations.

It is very difficult to evaluate the extent of patient selection in different patient materials, although younger age usually indicates more selection since cancers such as CRC are a disease of the elderly. In mCRC, a higher proportion of right-sided colon tumors, more female patients, older age, and more patients with poor performance (and thus fewer patients actively treated) generally indicate less selection. The PRCRC-study and Uppsala region cohorts were created with the intent to minimize selection having a prospective design with efforts to include as many patients as possible and finally to retrospectively identify remaining patients *via* registries. The entire PRCRC-study and Uppsala region cohorts have, thus, probably identified all (100%) *in vivo* diagnosed cases with mCRC. The RAXO data collection has the same intention, but as data are not validated yet, only patients with *KRAS-*G12C tumors were reported with a nearly 100% capture in the four regions covering 62% of the Finnish population. For the molecular analyses, selection will inevitably be present since diagnostic materials sufficient for analysis will not always be obtained from the oldest patients and from those with a rapid clinical course. Lack of sufficient tissue for molecular analyses in mCRC is a significant negative prognostic marker (24). Even if a biopsy was aimed at, it was not infrequently insufficient for any analyses more than to confirm the diagnosis of invasive adenocarcinoma. If materials had been obtained from every individual (i.e., also the 17% with no molecular analyses), it is possible that *KRAS-*G12C had been even slightly less common and, conversely, *BRAF*-V600E and dMMR even more frequent.

A weakness of the study is that all molecular tests were done in clinical routine, however, as recommended by ESMO guidelines (33, 34). This meant that for patients included early in the Uppsala cohort and in the Finnish population cohort, rare *KRAS* mutations such as those in codons 117 and 146, *NRAS* mutations, and *BRAF* mutations were not covered. This does not invalidate the estimations of the proportions of *KRAS-*G12C among all patients with mCRC but slightly overestimates the proportion of *KRAS*-G12 mutations among those with any *KRAS* mutation. From a scientific point of view, the use of only one technique covering all relevant genes would have been an advantage. However, relying on testing performed in clinical routine could also be looked upon as a strength considering the purpose of the study to explore the presence and behavior of this mutation in relation to others in the background population.

It is legitimate to discuss the relevance of finding the true prevalence of a certain trait in the background population. When it comes to toxic treatments such as chemotherapy and many target drugs, the patients must be at least reasonably well to be treated. Newer drugs may be less toxic and have an entirely different toxicity profile, making it highly relevant to know the distribution also in elderly, frail, and poor-performance-status patients. Immunotherapy for dMMR may be an example of this. The specific drugs presently under development for this mutation may be another example, as, at least in the early phases of development, toxicity profiles are considered “favorable” (7, 8).

In conclusion, in these real-life and population-based cohorts reflecting the background population, we did not detect any significant or clinically relevant differences in patient characteristics and tumor outcomes between patients with *KRAS-*G12C tumors compared to those with other *KRAS* mutations. The similarities in OS between *KRAS-*G12C and other-*KRAS* mutant tumors were seen irrespective of if the patients were treated with cytotoxics only or in combination with biologic agents, treated with metastasectomy and/or LAT, or handled with BSC. This contrasts with the results of previous studies mostly claiming worse outcomes in *KRAS-*G12C patients. Since population-based studies, minimizing patient selection, are more relevant for studies exploring the natural course of any property than clinical trials are, it is our belief that *KRAS-*G12C tumors behave similarly to all other *KRAS*- (and *NRAS*-) mutated tumors. Clinical trials following specific treatment protocols are more relevant when exploring outcomes after specific treatments. We could not detect any indications that the mutations differed when homogeneous treatment groups were analyzed and, thus, also believe that prognosis is not different according to the treatments used today, although our conclusion is based upon a few patients in each treatment group, however, not fundamentally fewer than in most studies having noticed worse survival for the *KRAS*-G12C mutation group. When specific drugs are developed, as for this mutation, differences in outcome will hopefully emerge.

## Congresses

Presented in part at the European Society for Medical Oncology (ESMO) World Congress on Gastrointestinal Cancer, Barcelona, June 30–July 3, 2021.

## Data Availability Statement

The data collected for this study can be made available to others in de-identified form after all primary and secondary endpoints have been published, in the presence of a data transfer agreement, and if the purpose of use complies with Nordic legislation. Requests for data sharing including a proposal that must be approved by the steering committee should be directed to BG, bengt.glimelius@igp.uu.se and PO, pia.osterlund@helsinki.fi.

## Ethics Statement

The studies involving human participants were reviewed and approved by Ethical Board at Bergen, Helsinki, Odense, and Uppsala University Hospitals. The prospective patients/participants provided their written informed consent to participate in this study.

## Author Contributions

EO, AR, SK, TK, EH, TM, PH, RK, L-MS, JS, MK, AÅ, RR, HS, PP, LN, TSa, AL, MM, TSj, HI, BG, and PO comprised the steering committee and participated in all phases of the study, including the design or conduct of the study, analyses and interpretation of the data, preparation of the manuscript, and the decision to submit. All authors recruited patients or gathered data for the study. EO and PO did the statistical analyses.

## Funding

Finska Läkaresällskapet (2016, 2018, 2019, 2020, 2021, 2022), The Finnish Cancer Foundation (2019-2020, 2021, 2022-2023), The Competitive State Research Financing of the Expert Responsibility Area of Tampere, Turku, Helsinki, Oulu and Kuopio University Hospitals (2016, 2017, 2018, 2019, 2020, 2021, 2022), Tampere and Helsinki University Hospital Research Funds (Tukisäätiö 2019, 2020; OOO 2020), The Sigrid Jusélius Foundation (2017, 2021), and The Swedish Cancer Society (2016, 2019) have provided grants. The infrastructure of the RAXO-study, with blood sampling, database, and study nurses, was supported by pharmaceutical companies: Amgen (unrestricted grants, 2012-2020), Lilly (2012-2017), Merck KGaA (2012-2020), Roche Oy (2012-2020), Sanofi (2012-2017), and Servier (unrestricted grant, 2016-2020). Amgen also partly supported the NGS analysis performed in patients included in the Uppsala region cohort. The funding sources had no role in the design and conduct of the study, collection, analysis and interpretation of the data, or decision to submit the manuscript for publication.

## Conflict of Interest

EO, AR, SK, TK, EH, TM, PH, RK, L-MS, JS, MK, AÅ, RR, PP, TSa, AL, MM, HI, and PO report institutional research funding from Eli Lilly, Merck KGaA, Nordic Drugs, Roche Oy, and Sanofi or unrestricted grants from Amgen and Servier, during the conduct of the study. EO, LN, TSj, and BG report unrestricted grants from Amgen for molecular analysis in the Uppsala region cohort. EO, AR, SK, TK, EH, TM, PH, RK, L-MS, JS, MK, AÅ, RR, HS, PP, TSa, AL, MM, HI, and PO report grants, personal fees, or non-financial support from AbbVie, Amgen, Astra-Zeneca, Bayer, Celgene, Eli Lilly, Eisai, Erytech Pharma, Incyte, Fresenius, Jansen-Cilag, Merck, MSD, Nordic Drugs, Nutricia, Pierre-Fabre, Roche, Sanofi, Servier, Sobi, or Varian.

## Publisher’s Note

All claims expressed in this article are solely those of the authors and do not necessarily represent those of their affiliated organizations, or those of the publisher, the editors and the reviewers. Any product that may be evaluated in this article, or claim that may be made by its manufacturer, is not guaranteed or endorsed by the publisher.

## References

[1] Vogelstein B Papadopoulos N Velculescu VE Zhou S Diaz LA Jr. Kinzler KW. Cancer Genome Landscapes. Science (2013) 339(6127):1546–58. doi: 10.1126/science.1235122 PMC3749880 23539594

[2] Sorich MJ Wiese MD Rowland A Kichenadasse G McKinnon RA Karapetis CS. Extended RAS Mutations and Anti-EGFR Monoclonal Antibody Survival Benefit in Metastatic Colorectal Cancer: A Meta-Analysis of Randomized, Controlled Trials. Ann Oncol (2015) 26(1):13–21. doi: 10.1093/annonc/mdu378 25115304

[3] Van Cutsem E Lenz HJ Kohne CH Heinemann V Tejpar S Melezinek I. Fluorouracil, Leucovorin, and Irinotecan Plus Cetuximab Treatment and RAS Mutations in Colorectal Cancer. J Clin Oncol (2015) 33(7):692–700. doi: 10.1200/JCO.2014.59.4812 25605843

[4] Tran E Ahmadzadeh M Lu YC Gros A Turcotte S Robbins PF. Immunogenicity of Somatic Mutations in Human Gastrointestinal Cancers. Science (2015) 350(6266):1387–90. doi: 10.1126/science.aad1253 PMC7445892 26516200

[5] Hobbs GA Der CJ Rossman KL. RAS Isoforms and Mutations in Cancer at a Glance. J Cell Sci (2016) 129(7):1287–92. doi: 10.1242/jcs.182873 PMC4869631 26985062

[6] Stahler A Heinemann V Ricard I von Einem JC Giessen-Jung C Westphalen CB. Current Treatment Options in RAS Mutant Metastatic Colorectal Cancer Patients: A Meta-Analysis of 14 Randomized Phase III Trials. J Cancer Res Clin Oncol (2020) 146(8):2077–87. doi: 10.1007/s00432-020-03290-y PMC7324435 32561975

[7] Hong DS Fakih MG Strickler JH Desai J Durm GA Shapiro GI. KRAS(G12C) Inhibition With Sotorasib in Advanced Solid Tumors. N Engl J Med (2020) 383(13):1207–17. doi: 10.1056/NEJMoa1917239 PMC7571518 32955176

[8] Wang C Fakih M. Targeting KRAS in Colorectal Cancer. Curr Oncol Rep (2021) 23(3):28. doi: 10.1007/s11912-021-01022-0 33582927

[9] Smith G Bounds R Wolf H Steele RJ Carey FA Wolf CR. Activating K-Ras Mutations Outwith ‘Hotspot’ Codons in Sporadic Colorectal Tumours - Implications for Personalised Cancer Medicine. Br J Cancer (2010) 102(4):693–703. doi: 10.1038/sj.bjc.6605534 20147967 PMC2837563

[10] Vaughn CP Zobell SD Furtado LV Baker CL Samowitz WS. Frequency of KRAS, BRAF, and NRAS Mutations in Colorectal Cancer. Genes Chromosomes Cancer (2011) 50(5):307–12. doi: 10.1002/gcc.20854 21305640

[11] Modest DP Ricard I Heinemann V Hegewisch-Becker S Schmiegel W Porschen R. Outcome According to KRAS-, NRAS- and BRAF-Mutation as Well as KRAS Mutation Variants: Pooled Analysis of Five Randomized Trials in Metastatic Colorectal Cancer by the AIO Colorectal Cancer Study Group. Ann Oncol (2016) 27(9):1746–53. doi: 10.1093/annonc/mdw261 PMC4999563 27358379

[12] Serebriiskii IG Connelly C Frampton G Newberg J Cooke M Miller V. Comprehensive Characterization of RAS Mutations in Colon and Rectal Cancers in Old and Young Patients. Nat Commun (2019) 10(1):3722. doi: 10.1038/s41467-019-11530-0 31427573 PMC6700103

[13] Schirripa M Nappo F Cremolini C Salvatore L Rossini D Bensi M. KRAS G12C Metastatic Colorectal Cancer: Specific Features of a New Emerging Target Population. Clin Colorectal Cancer (2020) 19(3):219–25. doi: 10.1016/j.clcc.2020.04.009 32605718

[14] Araujo LH Souza BM Leite LR Parma SAF Lopes NP Malta FSV. Molecular Profile of KRAS G12C-Mutant Colorectal and Non-Small-Cell Lung Cancer. BMC Cancer (2021) 21(1):193. doi: 10.1186/s12885-021-07884-8 33632153 PMC7905642

[15] Henry JT Coker O Chowdhury S Shen JP Morris VK Dasari A. Comprehensive Clinical and Molecular Characterization of KRAS (G12C)-Mutant Colorectal Cancer. JCO Precis Oncol (2021) 5:613–21. doi: 10.1200/PO.20.00256 PMC8232253 34250391

[16] Chida K Kotani D Masuishi T Kawakami T Kawamoto Y Kato K. The Prognostic Impact of KRAS G12C Mutation in Patients with Metastatic Colorectal Cancer: A Multicenter Retrospective Observational Study. Oncologist (2021) 26(10):845–53. doi: 10.1002/onco.13870 PMC8488780 34232546

[17] Giampieri R Lupi A Ziranu P Bittoni A Pretta A Pecci F. Retrospective Comparative Analysis of KRAS G12C vs. Other KRAS Mutations in mCRC Patients Treated With First-Line Chemotherapy Doublet + Bevacizumab. Front Oncol (2021) 11:736104. doi: 10.3389/fonc.2021.736104 34660299 PMC8514824

[18] Li N Li D Du Y Su C Yang C Lin C. Overexpressed PLAGL2 Transcriptionally Activates Wnt6 and Promotes Cancer Development in Colorectal Cancer. Oncol Rep (2019) 41(2):875–84. doi: 10.3892/or.2018.6914 PMC6313070 30535429

[19] Patelli G Tosi F Amatu A Mauri G Curaba A Patane DA. Strategies to Tackle RAS-Mutated Metastatic Colorectal Cancer. ESMO Open (2021) 6(3):100156. doi: 10.1016/j.esmoop.2021.100156 34044286 PMC8167159

[20] Ottaiano A Normanno N Facchini S Cassata A Nappi A Romano C. Study of Ras Mutations’ Prognostic Value in Metastatic Colorectal Cancer: STORIA Analysis. Cancers (Basel) (2020) 12(7):1919. doi: 10.3390/cancers12071919 PMC7409181 32708575

[21] Neumann J Zeindl-Eberhart E Kirchner T Jung A. Frequency and Type of KRAS Mutations in Routine Diagnostic Analysis of Metastatic Colorectal Cancer. Pathol Res Pract (2009) 205(12):858–62. doi: 10.1016/j.prp.2009.07.010 19679400

[22] Gil Ferreira C Aran V Zalcberg-Renault I Victorino AP Salem JH Bonamino MH. KRAS Mutations: Variable Incidences in a Brazilian Cohort of 8,234 Metastatic Colorectal Cancer Patients. BMC Gastroenterol (2014) 14:73. doi: 10.1186/1471-230X-14-73 24720724 PMC3997472

[23] Modest DP Brodowicz T Stintzing S Jung A Neumann J Laubender RP. Impact of the Specific Mutation in KRAS Codon 12 Mutated Tumors on Treatment Efficacy in Patients With Metastatic Colorectal Cancer Receiving Cetuximab-Based First-Line Therapy: A Pooled Analysis of Three Trials. Oncology (2012) 83(5):241–7. doi: 10.1159/000339534 22948721

[24] Sorbye H Dragomir A Sundstrom M Pfeiffer P Thunberg U Bergfors M. High BRAF Mutation Frequency and Marked Survival Differences in Subgroups According to KRAS/BRAF Mutation Status and Tumor Tissue Availability in a Prospective Population-Based Metastatic Colorectal Cancer Cohort. PloS One (2015) 10(6):e0131046. doi: 10.1371/journal.pone.0131046 26121270 PMC4484806

[25] Aasebo KO Dragomir A Sundstrom M Mezheyeuski A Edqvist PH Eide GE. Consequences of a High Incidence of Microsatellite Instability and BRAF-Mutated Tumors: A Population-Based Cohort of Metastatic Colorectal Cancer Patients. Cancer Med (2019) 8(7):3623–35. doi: 10.1002/cam4.2205 PMC6601706 31070306

[26] Nunes L Aasebo K Mathot L Ljungstrom V Edqvist PH Sundstrom M. Molecular Characterization of a Large Unselected Cohort of Metastatic Colorectal Cancers in Relation to Primary Tumor Location, Rare Metastatic Sites and Prognosis. Acta Oncol (2020) 59(4):417–26. doi: 10.1080/0284186X.2019.1711169 31924107

[27] Osterlund P Salminen T Soveri LM Kallio R Kellokumpu I Lamminmaki A. Repeated Centralized Multidisciplinary Team Assessment of Resectability, Clinical Behavior, and Outcomes in 1086 Finnish Metastatic Colorectal Cancer Patients (RAXO): A Nationwide Prospective Intervention Study. Lancet Reg Health Eur (2021) 3:100049. doi: 10.1016/j.lanepe.2021.100049 34557799 PMC8454802

[28] Isoniemi H Uutela A Nordin A Lantto E Kellokumpu I Ovissi A. Centralized Repeated Resectability Assessment of Patients With Colorectal Liver Metastases During First-Line Treatment: Prospective Study. Br J Surg (2021) 108(7):817–25. doi: 10.1093/bjs/znaa145 PMC10364914 33749772

[29] Sorbye H Pfeiffer P Cavalli-Bjorkman N Qvortrup C Holsen MH Wentzel-Larsen T. Clinical Trial Enrollment, Patient Characteristics, and Survival Differences in Prospectively Registered Metastatic Colorectal Cancer Patients. Cancer (2009) 115(20):4679–87. doi: 10.1002/cncr.24527 19562777

[30] Glimelius B Melin B Enblad G Alafuzoff I Beskow A Ahlstrom H. U-CAN: A Prospective Longitudinal Collection of Biomaterials and Clinical Information From Adult Cancer Patients in Sweden. Acta Oncol (2018) 57(2):187–94. doi: 10.1080/0284186X.2017.1337926 28631533

[31] Pahlman L Bohe M Cedermark B Dahlberg M Lindmark G Sjodahl R. The Swedish Rectal Cancer Registry. Br J Surg (2007) 94(10):1285–92. doi: 10.1002/bjs.5679 17661309

[32] Osterman E Hammarstrom K Imam I Osterlund E Sjoblom T Glimelius B. Completeness and Accuracy of the Registration of Recurrences in the Swedish Colorectal Cancer Registry (SCRCR) and an Update of Recurrence Risk in Colon Cancer. Acta Oncol (2021) 60(7):842–9. doi: 10.1080/0284186X.2021.1896033 33689551

[33] Schmoll HJ Van Cutsem E Stein A Valentini V Glimelius B Haustermans K. ESMO Consensus Guidelines for Management of Patients With Colon and Rectal Cancer. A Personalized Approach to Clinical Decision Making. Ann Oncol (2012) 23(10):2479–516. doi: 10.1093/annonc/mds236 23012255

[34] Van Cutsem E Cervantes A Adam R Sobrero A Van Krieken JH Aderka D. ESMO Consensus Guidelines for the Management of Patients With Metastatic Colorectal Cancer. Ann Oncol (2016) 27(8):1386–422. doi: 10.1093/annonc/mdw235 27380959

[35] Sundstrom M Edlund K Lindell M Glimelius B Birgisson H Micke P. KRAS Analysis in Colorectal Carcinoma: Analytical Aspects of Pyrosequencing and Allele-Specific PCR in Clinical Practice. BMC Cancer (2010) 10:660. doi: 10.1186/1471-2407-10-660 21122130 PMC3002357

[36] Salem M El-Refai S Sha W Grothey A Puccini A George T. Characterization of KRAS Mutation Variants and Prevalence of KRAS-G12C in Gastrointestinal Malignancies. Ann Oncol (2021) 32:S218. doi: 10.1016/j.annonc.2021.05.007

[37] Riely GJ Kris MG Rosenbaum D Marks J Li A Chitale DA. Frequency and Distinctive Spectrum of KRAS Mutations in Never Smokers With Lung Adenocarcinoma. Clin Cancer Res (2008) 14(18):5731–4. doi: 10.1158/1078-0432.CCR-08-0646 PMC2754127 18794081

[38] Dogan S Shen R Ang DC Johnson ML D’Angelo SP Paik PK. Molecular Epidemiology of EGFR and KRAS Mutations in 3,026 Lung Adenocarcinomas: Higher Susceptibility of Women to Smoking-Related KRAS-Mutant Cancers. Clin Cancer Res (2012) 18(22):6169–77. doi: 10.1158/1078-0432.CCR-11-3265 PMC3500422 23014527

[39] Algars A Sundstrom J Lintunen M Jokilehto T Kytola S Kaare M. EGFR Gene Copy Number Predicts Response to Anti-EGFR Treatment in RAS Wild Type and RAS/BRAF/PIK3CA Wild Type Metastatic Colorectal Cancer. Int J Cancer (2017) 140(4):922–9. doi: 10.1002/ijc.30507 27879995

[40] Aasebo K Dragomir A Sundstrom M Mezheyeuski A Edqvist PH Eide GE. CDX2: A Prognostic Marker in Metastatic Colorectal Cancer Defining a Better BRAF Mutated and a Worse KRAS Mutated Subgroup. Front Oncol (2020) 10:8. doi: 10.3389/fonc.2020.00008 32117703 PMC7026487

