# Supplementary material for: KRAS-G12C Mutation in One Real-Life and Three Population-Based Nordic Cohorts of Metastatic Colorectal Cancer
Source: Front Oncol. 2022 Feb 16;12:826073. doi: 10.3389/fonc.2022.826073 (PMC8889930; doi:10.3389/fonc.2022.826073)
Supplement: Supplementary file 1 [file DataSheet_1.pdf]

Supplementary Material

Supplementary Table 1. Demographics for the different cohorts included

		All∫		Real-life RAXO 2012-2018		Uppsala region 2010-2018		PRCRC 2003-2006		p-value*
		2559	100 %	1086	44 %	626	26 %	798	33 %	
Total		2559	100 %	1086	100 %	626	100 %	798	100 %	
Age groups	≤70	1386	54 %	716	66 %	264	42 %	376	47 %	<0.001
	>70	1173	46 %	370	34 %	362	58 %	422	53 %	
Sex	Male	1430	56 %	656	60 %	331	53 %	418	52 %	0.002
	Female	1129	44 %	430	40 %	295	47 %	380	48 %	
Primary tumor	Right colon	839	33 %	310	29 %	235	38 %	278	36 %	<0.001
	Left colon	836	33 %	396	37 %	172	28 %	252	33 %	
	Rectum	851	34 %	374	35 %	215	35 %	245	32 %	
	Multiple/unknown	33	-	6	-	4	-	23	-	
Primary surgery	No	756	30 %	250	23 %	272	44 %	223	28 %	<0.001
	Yes	1803	71 %	836	77 %	354	57 %	575	72 %	
Presentation of metastases	Synchronous	1618	63 %	735	68 %	422	68 %	433	54 %	<0.001
	Metachronous	305	12 %	351	32 %	203	32 %	365	46 %	
ECOG Performance status	0	765	30 %	295	27 %	208	33 %	250	31 %	<0.001
	1	1053	41 %	600	55 %	193	31 %	232	29 %	
	2-4	732	29 %	191	18 %	220	35 %	313	39 %	
	Missing	9	-	0	-	5	-	3	-	
Type of treatment	Any active treatment	2011	79 %	1063	98 %	443	71 %	460	58 %	<0.001
	Metastasectomy and/or LAT ± systemic therapy	626	25 %	397	37 %	159	25 %	49	6 %	<0.001
	Systemic therapy only	1385	54 %	666	61 %	284	45 %	411	52 %	<0.001
	Cytotoxics only	563	22 %	117	11 %	175	28 %	293	37 %	<0.001
	Anti-VEGF/Anti-EGFR§	822	32 %	549	51 %	138	22 %	118	15 %	<0.001
	Best supportive care	548	21 %	23	2 %	183	29 %	338	42 %	<0.001
KRAS, NRAS, BRAF testing	Not sufficiently	688	27 %	167	15 %	175	28 %	346	43 %	<0.001
	Accurately analyzed	1871	73 %	919	85 %	451	72 %	452	57 %	
Overall survival	Median, months (95% CI)	18.9 (17.8-20.1)		30.0 (28.0-32.1)		15.3 (13.3-17.2)		9.8 (8.5-11.0)		<0.001
	1-year rate	64 %		82 %		58 %		43 %		
	3-year rate	28 %		42 %		24 %		11 %		
	Any active treatment	25.2 (23.8-26.5)		30.6 (28.4-32.8)		22.8 (20.6-25.0)		16.2 (14.4-18.1)		<0.001
	Metastasectomy and/or LAT	67.5 (61.3-73.8)		71.5 (62.8-80.2)		60.5 (51.1-70.0)		61.4 (47.9-74.8)		0.328
	Systemic therapy only	18.0 (16.9-19.0)		20.8 (19.3-22.3)		15.0 (13.8-16.1)		15.1 (13.4-16.8)		<0.001
	Cytotoxics only	11.3 (10.4-12.3)		10.8 (7.7-13.8)		11.9 (10.4-13.3)		10.9 (9.1-12.7)		0.132
	Anti-VEGF/Anti-EGFR §	23.0 (21.5-24.5)		23.2 (21.2-25.2)		23.9 (20.0-27.8)		20.7 (17.3-24.1)		0.782
	Best supportive care	3.4 (3.0-3.8)		2.9 (2.6-3.1)		3.2 (2.7-3.7)		4.6 (3.3-5.8)		0.009

§ systemic therapy with anti-VEGF/anti-EGFR but without metastasectomy/LAT.
* p-value between RAXO, Uppsala Region and PRCRC.
∫ Includes 49 patients from the Finnish population cohort (characteristics presented in Supplementary Table 2. Characteristics of all patients in the Finnish population cohort are not detailed as in the three cohorts presented above).

Supplementary Table 2. Demographics for molecularly adequately analyzed tumors in the different cohorts included

		All		RAXO		Uppsala region		PRCRC		p-value	*KRAS*-G12Cmt in Finnish data-collection	
		1871	100 %	919	49 %	451	24 %	452	24 %		49	3 %
Total		1871	100 %	919	100 %	451	100 %	452	100 %		49	100 %
Mutation groups	*KRAS*-G12Cmt	103	6 %	34	4 %	12	3 %	8	2 %	<0.001	49	100 %
	*KRAS*mt	881	47 %	461	50 %	223	49 %	197	44 %		0	0 %
	*NRAS*mt	66	4 %	37	4 %	12	3 %	17	4 %		0	0 %
	RAS&*BRAF*wt	548	29 %	294	32 %	113	25 %	141	31 %		0	0 %
	*BRAF*V600Emt	273	15 %	93	10 %	91	20 %	89	20 %		0	0 %
Agegroups	≤70	1074	57 %	610	66 %	200	44 %	234	52 %	<0.001	30	61 %
	>70	797	43 %	309	34 %	251	56 %	218	48 %		19	39 %
Sex	Male	1053	56 %	558	61 %	245	54 %	225	50 %	<0.001	25	51 %
	Female	818	44 %	361	39 %	206	46 %	227	50 %		24	49 %
Primary tumor	Right colon	624	34 %	267	29 %	167	37 %	174	38 %	<0.001	16	33 %
	Left colon	619	33 %	329	36 %	117	26 %	157	35 %		16	33 %
	Rectum	612	33 %	318	35 %	164	37 %	113	25 %		17	35 %
	Multiple/unknown	16	-	5	-	3	-	8	-		0	-
Primary surgery	No	449	24 %	215	23 %	183	41 %	40	9 %	<0.001	11	22 %
	Yes	1422	76 %	704	77 %	268	59 %	412	91 %		38	78 %
Presentation of metastases	Synchronous	1146	61 %	622	68 %	292	65 %	205	45 %	<0.001	28	57 %
	Metachronous	229	12 %	297	32 %	159	35 %	247	55 %		21	43 %
Performance status	0	593	32 %	256	28 %	161	36 %	164	36 %	<0.001	12	25 %
	1	815	44 %	507	55 %	141	32 %	139	31 %		28	58 %
	2-4	457	25 %	156	17 %	145	32 %	148	33 %		8	17 %
	Missing	6	-	0	-	4	-	1	-		1	-
Type of treatment	Any active treatment	1593	85 %	902	98 %	354	79 %	292	65 %	<0.001	45	92 %
	Metastasectomy and/or LAT ± systemic therapy	516	28 %	337	37 %	122	27 %	36	8 %	<0.001	21	43 %
	Systemic therapy only	1077	58 %	565	61 %	232	51 %	256	57 %	<0.001	24	49 %
	Cytotoxics only	406	22 %	94	10 %	120	27 %	185	41 %	<0.001	7	14 %
	Anti-VEGF/Anti-EGFR§	671	36 %	471	51 %	112	25 %	71	16 %	<0.001	17	35 %
	Best supportive care	278	15 %	17	2 %	97	22 %	160	35 %	<0.001	4	8 %
Overall survival	Median, months (95% CI)	22.1 (20.9-23.5)		30.4 (28.0-32.9)		17.7 (15.1-20.2)		11.9 (10.2-13.6)		<0.001	31.6 (21.3-41.9)	
	1-year rate	70 %		84 %		62 %		50 %			76 %	
	3-year rate	32 %		43 %		26 %		14 %			41 %	
	Any active treatment	26.5 (24.9-28.1)		31.0 (28.5-33.5)		22.9 (20.4-25.3)		18.0 (15.4-20.6)		<0.001	33.0 (31.1-34.8)	
	Metastasectomy and/or LAT	67.5 (60.7-74.3)		71.8 (61.9-81.7)		58.5 (42.9-74.1)		64.0 (19.9-108.1)		0.117	54.5 (30.2-78.8)	
	Systemic therapy only	18.9 (17.8-20.1)		21.1 (19.5-22.8)		15.1 (13.4-16.7)		15.8 (14.4-17.2)		<0.001	20.9 (13.5-28.2)	
	Cytotoxics only	12.4 (11.1-13.7)		13.7 (11.1-16.4)		11.0 (9.5-12.6)		12.5 (10.2-14.8)		0.036	6.9 (4.4-9.3)	
	Anti-VEGF/Anti-EGFR §	23.5 (21.7-25.2)		23.2 (21.0-25.4)		20.7 (16.6-24.9)		26.9 (22.2-31.5)		0.898	24.7 (20.6-28.8)	
	Best supportive care	4.2 (3.5-5.0)		2.9 (2.1-3.7)		4.8 (2.5-7.0)		4.1 (3.1-5.2)		0.170	3.3 (2.3-4.4)	

§ systemic therapy with anti-VEGF/anti-EGFR but without metastasectomy/LAT.

*p-value between RAXO, Uppsala region and PRCRC, § systemic therapy with anti-VEGF/anti-EGFR but without metastasectomy/LAT. The high percentage of *KRAS*-G12C mutations among all patients depend upon the enrichment of 49 patients with this mutation from the Finnish population cohort.

Supplementary Table 3. Smoking status, resection of primary tumor, blood counts, alkaline phosphatase (ALP) and carcinoembryonic antigen (CEA) per mutation group

		All		*KRAS*-G12C mt		Other *KRAS* mt		*NRAS* mt		*RAS* & *BRAF* wt		*BRAF* V600E mt		p-value*
		1871	100 %	103	100 %	881	100 %	66	100 %	548	100 %	273	100 %	
Smoking status	Never	711	53 %	40	63 %	341	54 %	29	57 %	220	53 %	81	43 %	ref
	Ex-smoker	303	23 %	10	16 %	135	22 %	12	24 %	96	23 %	50	26 %	0.211
	Smoker	332	25 %	14	22 %	153	24 %	10	20 %	96	23 %	59	31 %	0.445
	Missing	525	-	39	-	252	-	15	-	136	-	83	-	-
Primary resection	No	449	24 %	23	22 %	226	26 %	17	26 %	117	21 %	66	24 %	ref
	Yes	1422	76 %	80	78 %	655	74 %	49	74 %	431	79 %	207	76 %	0.464
Haemoglobin	≥11.0	1232	83 %	74	82 %	572	82 %	47	84 %	381	84 %	158	81 %	ref
	<11.0	258	17 %	16	18 %	124	18 %	9	16 %	73	16 %	36	19 %	0.993
	Missing	381	-	13	-	185	-	10	-	94	-	79	-	-
Histology	Adenocarcinoma	1067	0.87	77	0.865	507	0.87	42	0.913	331	0.912	110	0.753	ref
	Mucinous adenocarcinoma	160	0.13	12	0.135	76	0.13	4	0.087	32	0.088	36	0.247	0.907
	Missing	644	-	14	-	298	-	20	-	185	-	127	-	-
Leukocytes	≤10.0	1175	79 %	73	81 %	561	81 %	41	75 %	364	80 %	136	70 %	ref
	>10.0	310	21 %	17	19 %	133	19 %	14	25 %	89	20 %	57	30 %	0.950
	Missing	386	-	13	-	187	-	11	-	95	-	80	-	-
Thrombocytes	≤400	1075	74 %	66	73 %	501	74 %	33	60 %	348	79 %	127	69 %	ref
	<400	370	26 %	24	27 %	172	26 %	22	40 %	94	21 %	58	31 %	0.821
	Missing	426	-	13	-	208	-	11	-	106	-	88	-	-
ALP	≤ULN	871	60 %	57	63 %	415	62 %	29	54 %	265	60 %	105	57 %	ref
	>ULN	572	40 %	33	37 %	256	38 %	25	46 %	178	40 %	80	43 %	0.785
	Missing	428	-	13	-	210	-	12	-	105	-	88	-	-
CEA	≤5	364	29 %	32	37 %	162	27 %	15	28 %	109	28 %	46	31 %	ref
	>5	913	71 %	55	63 %	440	73 %	38	72 %	278	72 %	102	69 %	0.057
	Missing	594	-	16	-	279	-	13	-	161	-	125	-	-

ULN=upper limit of normal

*p-value between *KRAS*-G12C mt and Other *KRAS* mt

Supplementary Table 4. Demographics per mutation group excluding the 49 patients with *KRAS*-G12C mutations from the Finnish population-based cohort not included in the real-life RAXO study

		All		*KRAS*-G12C mt		Other *KRAS* mt		*NRAS* mt		*RAS* & *BRAF* wt		*BRAF* V600E mt		p-value*
Total		1822	100 %	54	100 %	881	100 %	66	100 %	548	100 %	273	100 %	
Median age (range)		68	(22-99)	68	(35-86)	69	(23-99)	64	(30-87)	67	(22-95)	70	(33-90)	0.215
Agegroups	≤70	1044	57 %	33	61 %	484	55 %	44	67 %	350	64 %	137	50 %	ref
	>70	778	43 %	21	39 %	397	45 %	22	33 %	198	36 %	136	50 %	0.377
Sex	Female	794	44 %	22	41 %	378	43 %	28	42 %	198	36 %	168	62 %	ref
	Male	1028	56 %	32	59 %	503	57 %	38	58 %	350	64 %	105	38 %	0.755
Performance status	0	581	32 %	15	28 %	297	34 %	25	38 %	176	32 %	68	25 %	ref
	1	787	43 %	22	41 %	394	45 %	31	47 %	241	44 %	99	37 %	0.770
	2-4	449	25 %	17	31 %	188	21 %	10	15 %	130	24 %	104	38 %	0.112
	Missing	5	-	0	-	2	-	0	-	1	-	2	-	-
Smoking status	Never	698	53 %	27	68 %	341	54 %	29	57 %	220	53 %	81	43 %	ref
	Ex-smoker	299	23 %	6	15 %	135	22 %	12	24 %	96	23 %	50	26 %	0.212
	Smoker	325	25 %	7	18 %	153	24 %	10	20 %	96	23 %	59	31 %	0.208
	Missing	500	-	14	-	252	-	15	-	136	-	83	-	-
Primary tumour	Right colon	608	34 %	13	24 %	295	34 %	9	14 %	95	17 %	196	73 %	ref
	Left colon	603	33 %	20	37 %	266	30 %	26	39 %	241	44 %	50	19 %	0.145
	Rectum	595	33 %	21	39 %	312	36 %	31	47 %	207	38 %	24	9 %	0.242
	Multiple/unknown	16	-	0	-	8	-	0	-	5	-	3	-	-
Primary resection	No	438	24 %	12	22 %	226	26 %	17	26 %	117	21 %	66	24 %	ref
	Yes	1384	76 %	42	78 %	655	74 %	49	74 %	431	79 %	207	76 %	0.575
Differentiation	Well/Moderate	1157	78 %	37	86 %	588	84 %	47	86 %	363	81 %	122	53 %	ref
	Poor/Undifferentiated	318	22 %	6	14 %	111	16 %	8	15 %	85	19 %	108	47 %	0.737
	Missing	347	-	11	-	182	-	11	-	100	-	43	-	-
Histology	Adenocarcinoma	1026	87 %	36	88 %	507	87 %	42	91 %	331	91 %	110	75 %	ref
	Mucinous adenocarcinoma	153	13 %	5	12 %	76	13 %	4	9 %	32	9 %	36	25 %	0.877
	Missing	643	-	13	-	298	-	20	-	185	-	127	-	-
Presentation of metastases	Synchronous	1119	61 %	33	61 %	555	63 %	41	62 %	318	58 %	172	63 %	ref
	Metachronous	703	39 %	21	39 %	326	37 %	25	38 %	230	42 %	101	37 %	0.781
Number of metastatic sites	1	890	49 %	28	52 %	443	50 %	31	47 %	263	48 %	125	46 %	ref
	2	600	33 %	18	33 %	287	33 %	22	33 %	178	32 %	95	35 %	0.980
	3+	332	18 %	8	15 %	151	17 %	13	20 %	107	20 %	53	19 %	0.668
Metastatic sites	Liver	1262	69 %	39	72 %	615	70 %	51	77 %	412	75 %	145	53 %	0.707
	Lung	566	31 %	19	35 %	321	36 %	22	33 %	136	25 %	68	25 %	0.853
	Lymph nodes	486	27 %	12	22 %	199	23 %	18	27 %	152	28 %	105	38 %	0.950
	Peritoneum	379	21 %	6	11 %	169	19 %	11	17 %	104	19 %	89	33 %	0.146
	Bone	68	4 %	2	4 %	31	4 %	2	3 %	23	4 %	10	4 %	0.943
	Other	248	14 %	6	11 %	106	12 %	9	14 %	78	14 %	49	18 %	0.840
Haemoglobin	≥11.0	1193	83 %	35	83 %	572	82 %	47	84 %	381	84 %	158	81 %	ref
	<11.0	249	17 %	7	17 %	124	18 %	9	16 %	73	16 %	36	19 %	0.850
	Missing	380	-	12	-	185	-	10	-	94	-	79	-	-
Leukocytes	≤10.0	1134	79 %	32	76 %	561	81 %	41	75 %	364	80 %	136	70 %	ref
	>10.0	303	21 %	10	24 %	133	19 %	14	25 %	89	20 %	57	30 %	0.461
	Missing	385	-	12	-	187	-	11	-	95	-	80	-	-
Thrombocytes	≤400	1040	74 %	31	74 %	501	74 %	33	60 %	348	79 %	127	69 %	ref
	<400	357	26 %	11	26 %	172	26 %	22	40 %	94	21 %	58	31 %	0.927
	Missing	425	-	12	-	208	-	11	-	106	-	88	-	-
ALP	≤ULN	840	60 %	26	62 %	415	62 %	29	54 %	265	60 %	105	57 %	ref
	>ULN	555	40 %	16	38 %	256	38 %	25	46 %	178	40 %	80	43 %	0.994
	Missing	427	-	12	-	210	-	12	-	105	-	88	-	-
CEA	≤5	343	28 %	11	28 %	162	27 %	15	28 %	109	28 %	46	31 %	ref
	>5	886	72 %	28	72 %	440	73 %	38	72 %	278	72 %	102	69 %	0.860
	Missing	1229	593	15	-	279	-	13	-	161	-	125	-	-
MMR-status	pMMR	891	92 %	16	94 %	425	97 %	27	90 %	292	98 %	131	71 %	ref
	dMMR	77	8 %	1	6 %	12	3 %	3	10 %	7	2 %	54	29 %	0.458
	Missing	854	-	37	-	444	-	36	-	249	-	88	-	-

*p-value between *KRAS*-G12C mt and Other *KRAS* mt, ULN=upper limit of normal

Supplementary Table 5. Treatment per mutation group excluding the 49 patients with *KRAS*-G12C mutations from the Finnish population-based cohort not included in the real-life RAXO study

		All		*KRAS*-G12C mt		Other *KRAS* mt		p-value*	*NRAS* mt		*RAS* & *BRAF* wt		*BRAF* V600E mt	
Total		1822	100 %	54	100 %	881	100 %		66	100 %	548	100 %	273	100 %
Type of treatment	Systemic therapy	1053	58 %	27	50 %	510	58 %	ref	39	59 %	308	56 %	169	62 %
	Metastasectomy and/or LAT	495	27 %	18	33 %	246	28 %	0.303	22	33 %	180	33 %	29	11 %
	Best supportive care	274	15 %	9	17 %	125	14 %	0.439	5	8 %	60	11 %	75	27 %
Total chemotherapy		1514	100 %	44	100 %	743	100 %		59	100 %	476	100 %	192	100 %
Number of lines	1	610	40 %	17	39 %	291	39 %	ref	21	36 %	197	41 %	84	44 %
	2	422	28 %	16	36 %	204	27 %	0.413	14	24 %	115	24 %	73	38 %
	≥3	482	32 %	11	25 %	248	33 %	0.487	24	41 %	164	34 %	35	18 %
First line chemotherapy	5-FU	1476	97 %	43	98 %	729	98 %	0.855	57	97 %	462	97 %	185	96 %
	Oxaliplatin	891	59 %	26	59 %	453	61 %	0.804	33	56 %	268	56 %	111	58 %
	Irinotecan	400	26 %	12	27 %	165	22 %	0.435	17	29 %	153	32 %	53	28 %
	Bevacizumab	648	43 %	25	57 %	348	47 %	0.200	33	56 %	163	34 %	79	41 %
	anti-EGFR	163	11 %	0	0 %	12	2 %	NE	4	7 %	136	29 %	11	6 %
Best response in first line	PR/CR/NED	794	57 %	19	49 %	368	53 %	0.568	32	58 %	307	68 %	68	40 %
	SD	419	30 %	14	36 %	238	35 %	ref	14	25 %	97	21 %	56	33 %
	PD	191	14 %	6	15 %	83	12 %	"	9	16 %	48	11 %	45	27 %
	Missing	110	-	5	-	54	-	-	4	-	24	-	23	-
Chemotherapy all lines	5-FU	1482	98 %	44	100 %	731	98 %	NE	57	97 %	463	97 %	187	97 %
	Oxaliplatin	1160	77 %	34	77 %	581	78 %	0.948	47	80 %	355	75 %	143	74 %
	Irinotecan	1057	70 %	30	68 %	511	69 %	0.934	46	78 %	344	72 %	126	66 %
	Bevacizumab	832	55 %	31	70 %	437	59 %	0.130	37	63 %	235	49 %	92	48 %
	anti-EGFR	364	24 %	0	0 %	36	5 %	NE	10	17 %	289	61 %	29	15 %

*p-value between *KRAS*-G12C mt and Other *KRAS* mt, LAT=local ablative therapy, NE=No estimate

Supplementary Figure 1. Overall survival for *KRAS*-G12C mutation based on type of treatment

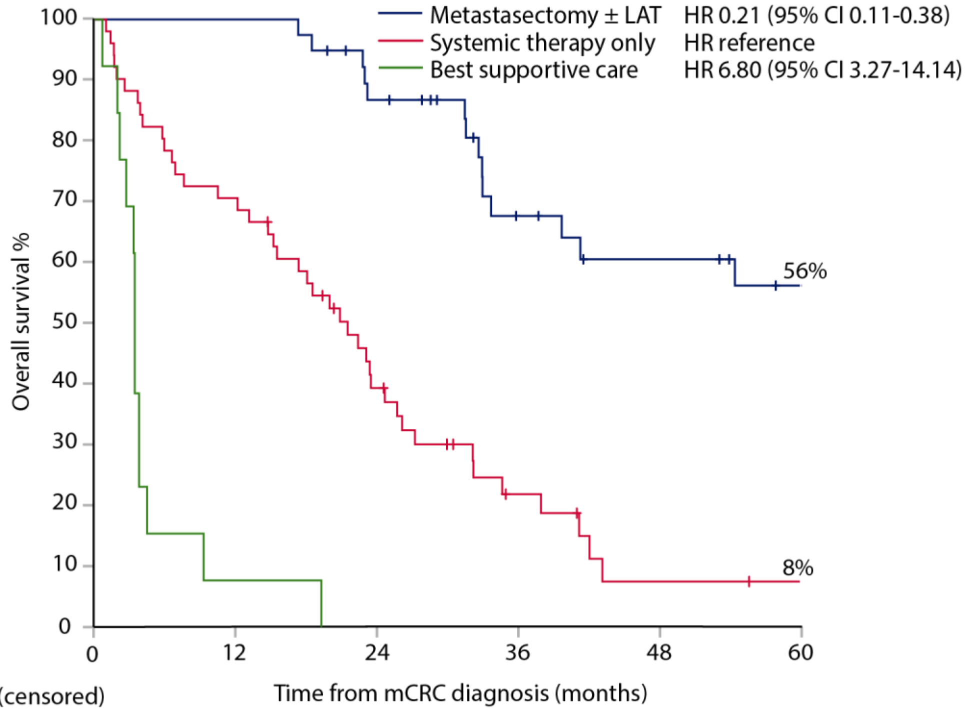

Number at risk (censored)

Surgery and/or LAT	39 (0)	39 (2)	32 (8)	20 (10)	16 (13)	12 (21)
Systemic therapy	51 (0)	36 (3)	18 (7)	7 (8)	2 (9)	1 (10)
Best supportive care	13 (0)	1 (0)	0 (0)	0 (0)	0 (0)	0 (0)

Supplementary Figure 2. Overall survival per group of mutation excluding the 49 patients with a *KRAS*-G12C mutated tumor from the Finnish population cohort: all patients (A), systemic therapy only (B), metastasectomy and/or local ablative therapy (C) and best supportive care (D)

A

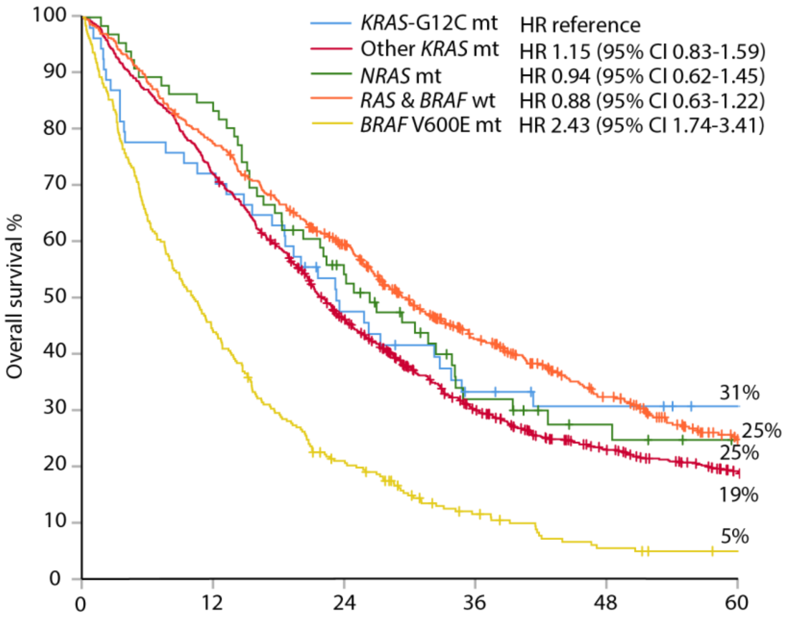

Number at risk (censored)

	0	12	24	36	48	60
KRAS-G12C mt	54 (0)	39 (2)	24 (4)	15 (6)	12 (9)	9 (15)
Other *KRAS* mt	881 (0)	635 (21)	389 (61)	221 (94)	140 (123)	89 (181)
NRAS mt	66 (0)	55 (3)	33 (8)	16 (12)	10 (15)	6 (20)
RAS & *BRAF* wt	548 (1)	424 (18)	310 (61)	185 (90)	116 (124)	59 (168)
BRAF V600E mt	273 (0)	120 (4)	54 (13)	23 (15)	10 (18)	6 (21)

B

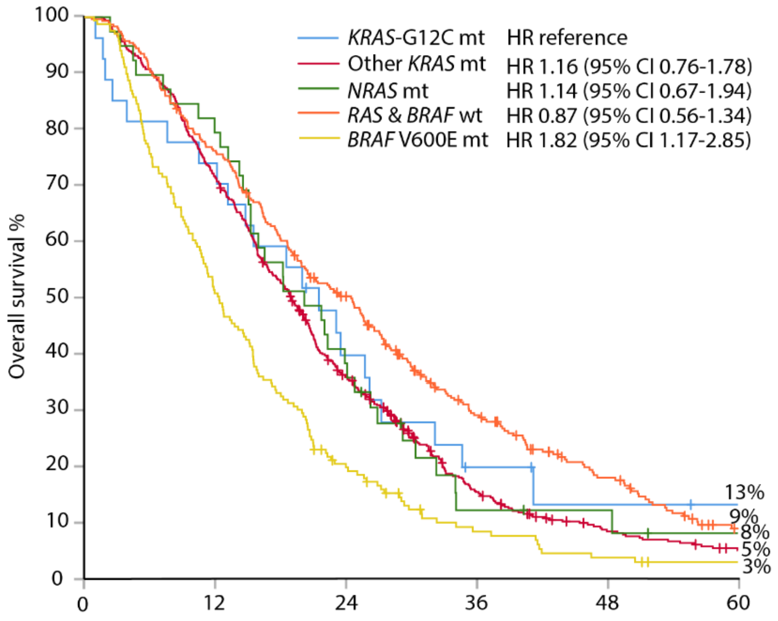

Number at risk (censored)

	0	12	24	36	48	60
KRAS-G12C mt	27 (0)	20 (1)	10 (2)	4 (3)	2 (4)	1 (5)
Other *KRAS* mt	510 (0)	365 (11)	175 (28)	66 (38)	29 (42)	14 (52)
NRAS mt	39 (0)	31 (0)	15 (2)	4 (3)	3 (4)	1 (4)
RAS & *BRAF* wt	308 (1)	234 (7)	149 (21)	76 (32)	39 (40)	13 (48)
BRAF V600E mt	169 (0)	86 (3)	32 (7)	11 (7)	5 (9)	2 (10)

C

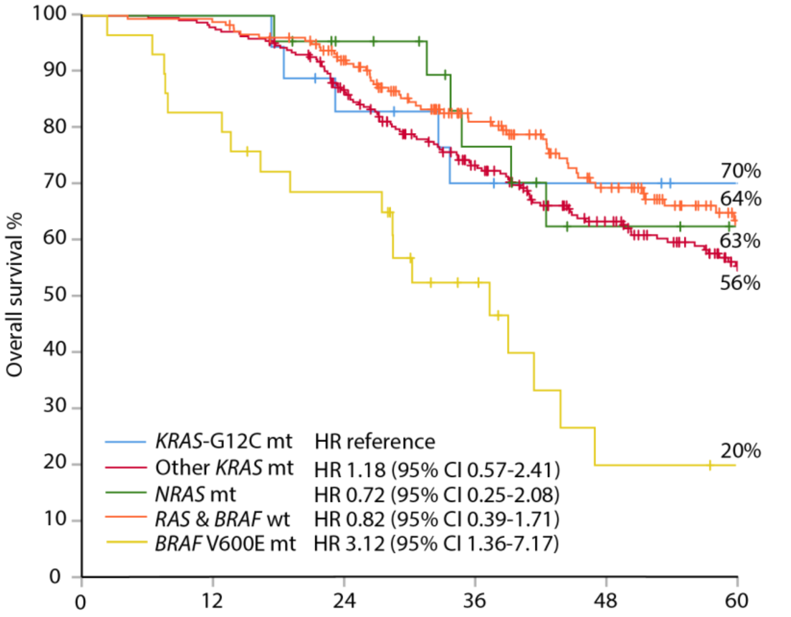

Number at risk (censored)

	0	12	24	36	48	60
KRAS-G12C mt	18 (0)	18 (1)	14 (2)	11 (3)	10 (5)	8 (10)
Other *KRAS* mt	246 (0)	241 (10)	204 (33)	151 (56)	109 (81)	73 (128)
NRAS mt	22 (0)	22 (3)	18 (6)	12 (9)	7 (11)	5 (16)
RAS & *BRAF* wt	180 (0)	178 (11)	155 (40)	109 (58)	77 (84)	46 (120)
BRAF V600E mt	29 (0)	24 (1)	19 (6)	10 (8)	3 (9)	2 (10)

D

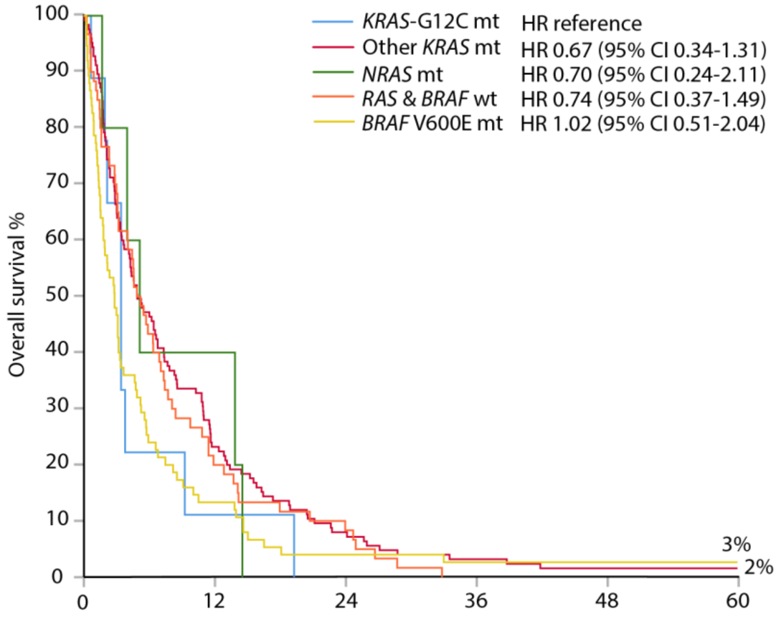

Number at risk (censored)

	0	12	24	36	48	60
KRAS-G12C mt	9 (0)	1 (0)	0 (0)	0 (0)	0 (0)	0 (0)
Other *KRAS* mt	125 (0)	29 (0)	10 (0)	4 (0)	2 (0)	2 (1)
NRAS mt	5 (0)	2 (0)	0 (0)	0 (0)	0 (0)	0 (0)
RAS & *BRAF* wt	60 (0)	12 (0)	6 (0)	0 (0)	0 (0)	0 (0)
BRAF V600E mt	75 (0)	10 (0)	3(0)	2 (0)	2 (0)	2 (1)

Supplementary Figure 3. Progression-free survival per group of mutation in patients treated with systemic therapy in all patients (A) and excluding the 49 patients with a *KRAS*-G12C mutated tumor from the Finnish population-based cohort (B)

A

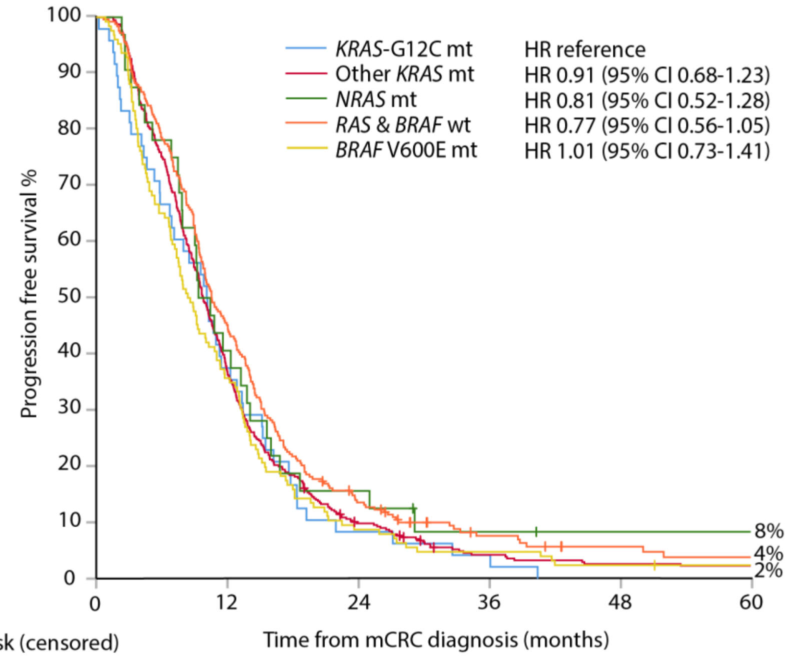

Number at risk (censored)

	0	12	24	36	48	60
KRAS-G12C mt	48 (0)	18 (0)	4 (0)	2 (0)	0 (0)	0 (0)
Other *KRAS* mt	391 (0)	145 (3)	36 (7)	13 (7)	8 (7)	7 (13)
NRAS mt	32 (0)	13 (0)	5 (1)	2 (2)	1 (2)	1 (2)
RAS & *BRAF* wt	248 (0)	112 (2)	32 (10)	12 (13)	6 (13)	4 (17)
BRAF V600E mt	126 (0)	45 (0)	11 (0)	6 (0)	3 (1)	2 (2)

B

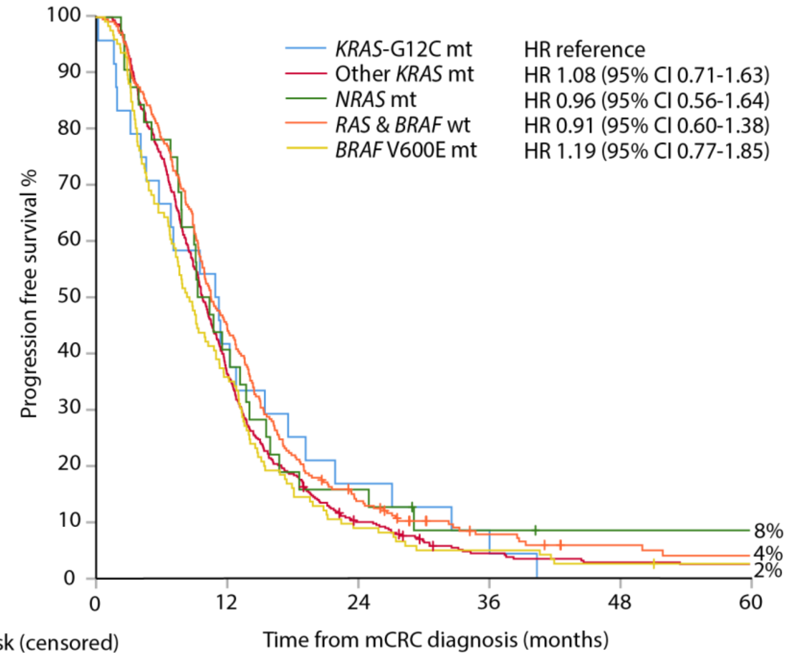

Number at risk (censored)

	0	12	24	36	48	60
KRAS-G12C mt	24 (0)	10 (0)	4 (0)	2 (0)	0 (0)	0 (0)
Other *KRAS* mt	391 (0)	145 (3)	36 (7)	13 (7)	8 (7)	7 (13)
NRAS mt	32 (0)	13 (0)	5 (1)	2 (2)	1 (2)	1 (2)
RAS & *BRAF* wt	248 (0)	112 (2)	32 (10)	12 (13)	6 (13)	4 (17)
BRAF V600E mt	126 (0)	45 (0)	11 (0)	6 (0)	3 (1)	2 (2)
